# Environmental risk factors for allergic rhinitis differ by income level: A Global Asthma Network survey

**DOI:** 10.1111/pai.70332

**Published:** 2026-04-10

**Authors:** Refiloe Masekela, Charlotte E. Rutter, Kevin Mortimer, Chen‐Yuan Chiang, Luis García‐Marcos, Asma El Sony, Karen Bissell, Eamon Ellwood, Neil Pearce, Innes Asher, Phillipa Ellwood, Nishtha Singh, Anele Khumalo, Eva Morales, the Global Asthma Network Phase I Study Group, Héctor Badellino, Dirceu Solé, Marilyn Urrutia‐Pereira, Achiri Elvis Ndikum, Manuel E Soto‐Martínez, Manuel E Soto‐Quirós, Angelita Cabrera Aguilar, Konstantinos Douros, Suyapa María Sosa Ferrari, Mohammed Sabir, Meenu Singh, Virendra Singh, Aloke Gopal Ghoshal, Thevaruparambil Unny Sukumaran, Shally Awasthi, Padukudru Anand Mahesh, Sushil Kumar Kabra, Sundeep Salvi, Marzieh Tavakol, Nasrin Behniafard, Shaker Abdulaziz Alomary, Wael A. Althagafi, Ibadete Bucaliu‐Ismajli, Laura Pajaziti, Valbona Gashi, Xhevat Kurhasani, Besa Gacaferri‐Lumezi, Luljeta Neziri Ahmetaj, Valbona Zhjeqi, MarÍa Guadalupe Sanchez Coronel, Héctor Leonardo Moreno Gardea, Georgina Ochoa‐Lopez, Roberto García‐Almaráz, José Antonio Sacre Hazouri, Noel Rodriguez‐Perez, Juan Valente Mérida‐Palacio, Blanca Estela Del Río Navarro, Luis Octavio Hernández‐Mondragón, Sandra Nora González‐Díaz, Rosa Garcia‐Muñoz, María de los Ángeles Juan Pineda, Beatriz del Carmen Ramos García, Alberton José Escalante‐Dominguez, Francisco Javier Linares‐Zapién, Elsy Maureen Navarrete‐Rodriguez, Jose Santos Lozano, José Félix Sánchez, Adegoke G. Falade, Grzegorz Brożek, Kseniiay Kyzmicheva, Heather J Zar, Angel López‐Silvarrey Varela, Carlos González Díaz, Alberto Bercedo Sanz, Javier Pellegrini Belinchon, Jagath Chaminda Ranasinghe, Sanath Thushara Kudagammana, Hana El Sadig, Magde Nour, Ghroob Alkhayer, Gazal Dib, Yousser Mohammad, Jing‐Long Huang, Sasawan Chinratanapisit, Pakit Vichyanond

**Affiliations:** ^1^ Department of Paediatrics and Child Health, College of Health Sciences, School of Clinical Medicine University of KwaZulu‐Natal Durban South Africa; ^2^ Africa Health Research Institute Durban South Africa; ^3^ Department of Medical Statistics London School of Hygiene & Tropical Medicine London UK; ^4^ Liverpool School of Tropical Medicine Liverpool UK; ^5^ Liverpool University Hospitals NHS Foundation Trust Liverpool UK; ^6^ University of Cambridge Cambridge UK; ^7^ International Union Against Tuberculosis and Lung Disease Paris France; ^8^ Division of Pulmonary Medicine, Department of Internal Medicine Wan Fang Hospital, Taipei Medical University Taipei Taiwan; ^9^ Division of Pulmonary Medicine, Department of Internal Medicine, College of Medicine School of Medicine, Taipei Medical University Taipei Taiwan; ^10^ Pediatric Allergy and Pulmonology Units Virgen de la Arrixaca University Children's Hospital, University of Murcia, and IMIB Bio‐Medical Research Institute, Edificio Departamental‐Laib Murcia Spain; ^11^ Epidemiological Laboratory (Epi‐Lab) for Public Health, Research and Development Khartoum Sudan; ^12^ School of Population Health, Faculty of Medical and Health Sciences University of Auckland Auckland New Zealand; ^13^ Department of Paediatrics: Child and Youth Health, Faculty of Medical and Health Sciences University of Auckland Auckland New Zealand; ^14^ Department of Respiratory Medicine Asthma Bhawan Jaipur India; ^15^ Department of Public Health Sciences, University of Murcia, and IMIB Bio‐Medical Research Institute, Edificio Departamental‐Laib Murcia Spain

**Keywords:** allergic rhinitis, exposure, low‐ to middle‐income countries, risk factors, rural

## Abstract

**Background:**

Estimating the prevalence and identifying risk factors for allergic rhinitis (AR) provides critical burden of disease data and offers opportunity to intervene in early‐life preventing morbidity.

**Methods:**

We conducted a Global Asthma Network (GAN) Phase I cross‐sectional study in children (6–7 years) and adolescents (13–14 years). Multilevel logistic regression models were fitted with random intercepts for school, center, and country, adjusting for sex and country income at the child level. Associations between symptoms and a range of lifestyle and environmental risk factors were assessed using odds ratios and corresponding 95% confidence intervals for mean individual and school exposure. Participants provided informed consent/assent, and each center was required to provide proof of ethical clearance.

**Results:**

We analysed data from 266,182 children and adolescents across 1688 schools in 65 centers for AR symptoms. Prevalence was 8.5% in children and 13.3% in adolescents. Early‐life exposures strongly associated with AR included paracetamol use (OR: 2.03; 95% CI: 1.89–2.18) and antibiotics (OR: 1.67; 95% CI: 1.56–1.78), with a stronger effect for antibiotics in low‐ and middle‐income countries (LMICs). Farm animal exposure increased AR risk among LMIC children (OR: 1.31; 95% CI: 1.12–1.53). In adolescents, computer use (OR: 1.28; 95% CI: 1.22–1.35) and tobacco use (OR: 1.37; 95% CI: 1.29–1.46) were significant risk factors. Heavy truck traffic consistently elevated AR risk in both age groups.

**Conclusion:**

The prevalence of AR is stable; early‐life exposures to animals increased the risk for AR in children from LMICs. Lifestyle factors and poor air quality from traffic‐related pollutants increase the risk of AR.

**Key message**

The Global Asthma Network (GAN) Phase I study examined the risk factors of allergic rhinitis in a global cohort of children and compared the association of these risk factors by country income level. We found a strong association between early‐life exposure to antibiotics and farm animals and allergic rhinitis in children living in low‐ and middle‐income countries. We found no association between early farm animal exposure and allergic rhinitis in children from high‐income countries. Truck traffic was consistently associated with allergic rhinitis, independent of country income level. For children in low‐ and middle‐income countries, liberal use of antibiotics and a more rural environment in the first year of life confer a risk of allergic rhinitis. Air pollution related to traffic is an important risk factor for allergic rhinitis, and interventions to reduce this could potentially reduce the burden of disease in both high‐income countries and low‐ and middle‐income countries.

## 1 INTRODUCTION

Allergic rhinitis (AR) is a common allergic disorder with an overall lifetime prevalence of up to 20% of the population, affecting between 10% and 25% of children.^1^, ^2^ Although AR is common and causes considerable costs to individuals suffering from the condition reflected in a poor quality of life and increased direct and indirect health care costs, it is poorly prioritised by health systems.^3^, ^4^ For children, nasal congestion a prominent symptom in AR is associated with sleep‐disordered breathing which can impact learning performance and behavior, and cause inattention. Other frequent co‐morbidities include asthma, otitis media, and sinusitis.

AR is a multifactorial disease most likely induced by gene–environment interactions.^5^ Exposures such as indoor and outdoor inhalant allergens can cause AR in individuals predisposed to an atopic phenotype. Identifying early‐life or current exposures that may increase the risk of AR, especially in children, could provide a crucial window for intervention to prevent or ameliorate the burden of disease in the population.

Previous studies have shown that interventions in early life which include diet and exposure prevention can impact future outcomes. Data from the Swedish BAMSE birth cohort study found that a higher intake of very long chain n3 and n6 fatty acids was protective against AR in young adulthood at age 24 years.^6^ The associations between early introduction of a diverse diet in infants and AR outcomes were not consistent, with some studies finding a protective effect between 6 and 12 months of life,^7^, ^8^ but a recent Healthy Start study^9^ did not find this association. Similarly, results on the impact of air pollution on AR outcomes in children and adults are inconsistent, with the RHINESSA study^10^ finding no association, while others have found an association between exposure to traffic‐related pollution in the last trimester of pregnancy and the first year of life.^11^

We therefore conducted the Global Asthma Network (GAN) Phase I study, with the aim to describe the prevalence of AR in school‐going children and further to analyze risk factors for AR in this global population.

## 2 METHODS

### 2.1 Study population

This GAN Phase I was formed by a collaboration of the International Study of Asthma and Allergies in Childhood (ISAAC) and the international union against tuberculosis and lung disease (Union). GAN continued the work of ISAAC, utilizing the standardized methodology developed by ISAAC to collect global data on the burden of asthma, AR, and eczema in school‐going children and adolescents^12^ This cross‐sectional epidemiological study was performed between 2015 and 2020. Written questionnaires were administered to children recruited in schools in two age groups: children (6–7 years) and adolescents (13–14 years) old. For children, the questionnaires were completed by parents or guardians, whereas for the adolescent age group they were self‐administered^12^

### 2.2 Definition of variables

We defined the main outcome of interest as the presence of AR based on a positive response to all of the following questions: (a) have you/this child ever had a problem with sneezing or a runny or blocked nose when you/this child did not have a cold or the flu? (b) in the past 12 months, have you/this child had a problem with sneezing or a runny or blocked nose when you/this child did not have a cold or the flu? and (c) in the past 12 months, has this nose problem been accompanied by itchy‐watery eyes? The exposure questions for the children (6–7 years) and adolescents (13–14 years) are shown in Tables S1 and S2.

For the children, the questionnaire data included questions on both prenatal and postnatal exposures. Categorical variables included prenatal exposure to animals (at least once a week contact with animals in pregnancy [yes]/[no]) and tobacco smoke (mother smoking during pregnancy [yes]/[no]). Postnatal events captured included low birth weight (<2.5 kg) and ever breastfeeding. The number of siblings was defined as either greater than one sibling or >2 siblings. For the early‐life events, contact with pets in the home was captured at two time points: either having a cat or dog in the house in the first year of life or in the past 12 months. Additionally, data on contact with farm animals, use of antibiotics and paracetamol use in the first year of life, and the frequency of truck traffic in the street and heavy truck traffic were collected. Lifestyle variables captured included the average intake of fast food, meat or burgers per week in the last 12 months and time spent watching television or using computers (including social media).

Most of the above risk factors were parameterized as binary variables from “yes/no.” The exceptions were: paracetamol use in the past 12 months (at least once per month vs. less than once per month), heavy truck traffic (frequently or almost the whole day vs. seldom or never), fast food consumption (once per week or more vs. less than once per week), television viewing (at least 1 h per day vs. <1 h per day), birth weight (<2.5 kg vs. at least 2.5 kg), and number of siblings (2 or more siblings vs. 1 or no siblings). For the adolescent age group, the questions were similar to those for 6–7 years olds except that, additionally, adolescents were asked about ever having smoked tobacco either daily or less than daily, in the past and early‐life questions were not asked.

The Gross National Income of the country was obtained from the World Bank website and countries were then divided into “affluent” or “non‐affluent” based on country income level using a gross national product per capita of $12, 535 USD for 2019 separating high‐income countries (HIC) and low‐ to middle‐income countries (LMICs).^13^

The data handling procedures have been described previously^12^ All participating centers submitted their datasets and were required to complete a center report (including sampling frame [schools, classes, and children], participation rates of schools and children, map of the area surveyed, rate of school rejection to participate and reasons why [if any], type of data entry and checking, how the questionnaire was translated, and dates of data collection) to the GAN Global Centre in Auckland, New Zealand, which performed initial data checks and an assessment of whether the center had adhered to the GAN methods. Depending on the language used by the center, the dataset was sent to Murcia, Spain (when the primary language of the center was Spanish or Portuguese) or London, UK (all other languages). A standardized and coordinated data check was performed, and a uniform approach to data processing, checking and analysis was used.

### 2.3 Statistical analysis

Separate analyses were conducted for the two age groups. Centers with fewer than 1000 individuals in an age group were excluded from the analyses for that age group. Each school was required to have at least 10 individuals to be included in the analyses for that age group. In addition, a response rate of at least 60% was required for children and at least 70% for adolescents for a center to be included.

Multilevel logistic regression models were fitted with random intercepts for school, center, and country and adjusted for sex at the child level and country affluence. Associations between AR symptoms and a range of lifestyle and environmental risk factors were assessed for both the child's exposure and mean exposure at the school using Odds ratios (OR) and their corresponding 95% confidence intervals (CI).

The potential risk factors for AR symptoms were compared at both the individual and school level.^14^, ^15^ Comparisons were drawn from GAN Phase I with the ISAAC Phase III study, where risk factors were adjusted for sex and country affluence or adjusted for sex, maternal level of education, paternal and maternal tobacco use, and open fire cooking, respectively.^14^, ^16^

Statistical analyses were conducted using STATA 17. StataCorp. 2021. Stata Statistical Software: Release 17. College Station, TX: StataCorp LLC. Each center was required to provide proof of ethical clearance for the study.

## 3 RESULTS

**FIGURE 1:**
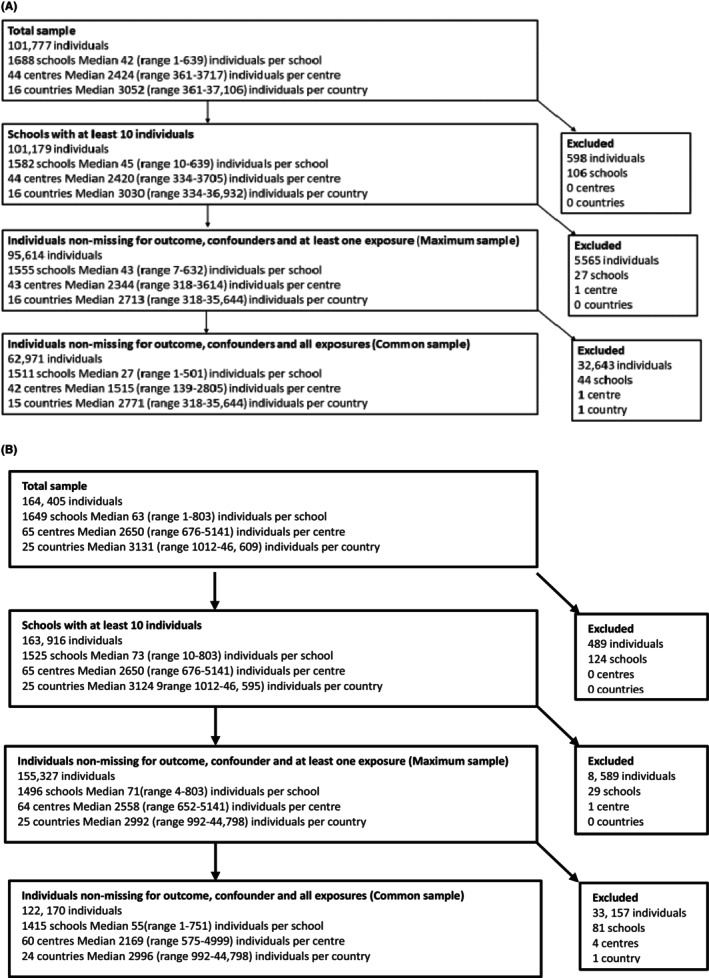
Data flow of the study population children (A) and adolescents (B).

A total of 266,182 children and adolescents from 1688 schools in 65 centers were screened for symptoms of AR. Of the children 6–7 years old, 101,777 children from 1688 schools in 44 centers were screened for AR symptoms (Figure 1A). Of these, 62,971 from 42 centers, 1511 schools and 15 countries were included in the final analysis (Figure 1A). For the adolescent age group, 164,405 individuals from 1649 schools in 65 centers were screened (Figure 1B). Of these, 122,170 were included for analysis from 60 centers and 24 countries (Table S3). The global individual‐level prevalence of AR was 8.5% for children and 13.3% for adolescents (Tables 1 and 2).

**TABLE 1:** Summary of prevalence, and individual‐ and school‐level exposures data for allergic rhinitis in children.

Variable	Individual level (*n* = 62,971)	School level (*n* = 1511)	
	Prevalence %	Median prevalence	IQR of prevalence
Rhinitis in past 12 months	8.5	6.3	(0.0, 12.5)
Animals in utero	6.1	4.1	(0.0, 9.5)
Smoking while pregnant	4.1	0.0	(0.0, 5.6)
Low birthweight	10.3	5.3	(0.0, 11.4)
Paracetamol when young	65.1	75.0	(50.0, 86.1)
Antibiotics when young	43.0	46.2	(30.8, 59.8)
Breastfed ever	87.8	88.7	(80.4, 95.1)
Cat when young	8.5	6.3	(0.0, 11.8)
Dog when young	22.0	20.0	(5.6, 35.0)
Animals when young	5.3	3.8	(0.0, 8.3)
More than 1 sibling	29.8	33.3	(18.2, 50.0)
More than 2 siblings	8.3	6.9	(1.4, 16.1)
Truck traffic	71.3	75.0	(61.5, 85.4)
Heavy truck traffic	29.9	26.3	(11.1, 48.1)
Fast food	27.9	25.0	(14.3, 37.5)
Fast food (excluding burgers)	27.7	24.4	(14.1, 37.5)
Meat (frequent)	37.4	24.4	(12.5, 50.0)
Television	74.0	77.4	(64.7, 86.2)
Computer	40.8	37.5	(25.0, 50.0)
Cat	13.5	9.8	(2.8, 52.3)
Dog	30.2	26.7	(9.7, 29.0)

**TABLE 2:** Summary of prevalence, and individual‐ and school‐level exposures data for adolescents.

Variable	Individual level (*n* = 122,170)	School level (*n* = 1415)	
	Prevalence %	Median prevalence	IQR of prevalence
Rhinitis in past 12 months	13.3	12.1	(6.9, 18.3)
More than 1 sibling	53.2	60.9	(39.4, 80.0)
More than 2 siblings	25.5	28.4	(11.7, 50.0)
Truck traffic	77.0	79.2	(67.2, 87.5)
Heavy truck traffic	32.9	29.3	(17.2, 42.9)
Fast food	45.8	43.8	(31.3, 60.0)
Fast food (excluding burgers)	46.9	46.5	(32.4, 63.2)
Meat (frequent)	31.1	29.4	(8.5, 46.8)
Television	72.9	72.3	(61.9, 80.0)
Computer	71.9	74.7	(41.7, 87.2)
Cat	23.1	19.0	(10.1, 31.2)
Dog	44.8	27.2	(10.3, 60.0)
Paracetamol	26.5	25.1	(16.7, 35.9)
Ever smoke	9.0	4.2	(0.0, 11.5)
Meat (frequent)	37.4	30.0	(12.5, 50.0)
Paracetamol	17.4	17.9	(9.7, 29.0)

### 3.1 Children school‐level risk factors

For the school‐level AR data, the median (interquartile range [IQR]) prevalence of AR was 6.3% (0.0; 12.5) (Table S4). With regard to prenatal exposures, 6.1% and 4.1% were exposed to animals or tobacco smoking in utero, respectively. The proportion who was ever breastfed was 87.7% and 10.3% were low birth weight. Over a third, 65.1%, had received paracetamol and 43% had received antibiotics when young. Early exposure to pets was observed in 22% for dogs and 8.5% for cats. Truck traffic exposure was in 71.3%, while almost a third, 29.9%, were exposed to heavy truck traffic. Over a quarter (27.7%) were eating fast foods regularly. Almost three‐quarters (74%) were watching more than an hour of television daily but a smaller proportion used computers (40.8%).

### 3.2 Adolescent school‐level risk factors

The school‐level median (IQR) prevalence of AR was 12.1% (6.9, 18.3) (Table 2). With regard to the individual‐level risk factors, over half (53.2%) had at least one sibling. A large proportion (77.0%) were exposed to truck traffic in the street, while almost a third (32.9%) were exposed to heavy truck traffic near their home. Almost half of the adolescents were eating fast foods at least once per week (45.8%), with the majority (72.9% and 71.9%) watching more than an hour of television or using computers for more than an hour, respectively. The proportion exposed to pets was 23.1% for cats and 44.8% for dogs. Almost a tenth (9.0%) reported ever smoking.

### 3.3 Multiple logistic regression of school‐level risk factors for children

After adjustment for sex and country affluence for all risk factors, use of paracetamol in early life increased the odds of AR independently of country affluence (OR = 2.03; 95% CI: 1.89, 2.18) (Table 3). This was similar for the school‐level data for paracetamol use OR: 2.11 (95% CI: 1.36, 3.26), and at the individual‐level OR: 2.03 (95% CI: 1.89, 2.18) (Tables S5 and S6).

**TABLE 3:** Individual‐level data on associations between exposure and allergic rhinitis in children.

Age 6–7, Exposure	GAN fully adjusted^a^		
	All (*n* = 62,971)	LMIC (*n* = 46,426)	HIC (*n* = 16,545)
	OR (95% CI)	OR (95% CI)	OR (95% CI)
Animals in utero	1.18 (1.04, 1.34)	1.22 (1.05, 1.42)	1.12 (0.88, 1.41)
Smoking while pregnant	1.16 (1.01, 1.33)	1.63 (1.31, 2.04)	0.99 (0.82, 1.18)
Low birthweight	1.08 (0.97, 1.21)	1.08 (0.93, 1.24)	1.09 (0.90, 1.32)
Paracetamol when young	1.13 (1.05, 1.22)	1.06 (0.95, 1.18)	1.22 (1.09, 1.36)
Antibiotics when young	1.67 (1.56, 1.78)	1.86 (1.71, 2.02)	1.38 (1.24, 1.54)
Breastfed ever	1.11 (1.01, 1.22)	1.05 (0.94, 1.18)	1.19 (1.02, 1.38)
Cat when young	1.06 (0.95, 1.19)	1.14 (1.00, 1.29)	0.84 (0.67, 1.07)
Dog when young	1.14 (1.06, 1.23)	1.19 (1.10, 1.30)	0.95 (0.80, 1.12)
Animals when young	1.24 (1.09, 1.42)	1.31 (1.12, 1.53)	1.11 (0.86, 1.44)
More than 1 sibling	0.91 (0.85, 0.98)	0.92 (0.85, 0.99)	0.88 (0.76, 1.01)
More than 2 siblings	NA	NA	NA
Truck traffic	NA	NA	NA
Heavy truck traffic	1.31 (1.23, 1.40)	1.35 (1.25, 1.46)	1.23 (1.09, 1.40)
Fast food	0.96 (0.89, 1.02)	0.93 (0.86, 1.01)	1.00 (0.89, 1.12)
Fast food (excluding burgers)	NA	NA	NA
Meat (frequent)	1.16 (1.09, 1.24)	1.28 (1.18, 1.39)	0.97 (0.87, 1.08)
Television	0.97 (0.90, 1.04)	0.97 (0.88, 1.06)	0.97 (0.86, 1.09)
Computer	1.09 (1.02, 1.16)	1.12 (1.03, 1.21)	1.03 (0.92, 1.15)
Cat	0.93 (0.84, 1.02)	0.92 (0.83, 1.03)	1.01 (0.81, 1.25)
Dog	0.98 (0.91, 1.06)	0.99 (0.91, 1.08)	1.01 (0.86, 1.19)
Paracetamol	2.03 (1.89, 2.18)	1.98 (1.83, 2.15)	2.17 (1.89, 2.51)

*Note*: NA: collinear with other variables not added to fully adjusted model.

^a^ Adjusted for sex and country income level.

At the school level, there was an association between early‐life exposure to antibiotics and AR (OR = 1.84; 95% CI: 1.23, 2.75). This was similar at the individual level with a significant (OR = 1.67; 95% CI: 1.56, 1.78), and this association was stronger in LMICs (OR = 1.86; 95% CI: 1.71, 2.02) compared with high‐income countries (HIC) (OR = 1.67; 95% CI: 1.56, 1.78).

Interestingly at the school level, a diet with increased meat intake was associated with higher odds of AR OR = 1.82 (95% CI: 1.28, 2.6) but this was again inconsistent across countries, reaching significance only in HICs. A diet with fast foods was not associated with an increased risk of AR either in children or adolescents; OR: 0.99 (95% CI: 0.94, 1.03) and OR: 1.04 (95% CI: 0.82, 1.32), respectively.

Use of computers showed an increased risk of AR overall (OR = 1.09; 95% CI: 1.02, 1.16) but this was inconsistent across countries, reaching significance in LMICs (OR = 1.12; 95% CI: 1.03, 1.21) but not HICs.

Exposure to animals in utero increased the risk of AR overall OR: 1.18 (95% CI: 1.04, 1.34) and this reached significance in LMIC countries OR: 1.22 (95% CI: 1.05, 1.42). There was an association between exposure to farm animals when young and AR 1.24 (95% CI: 1.09, 1.42), but this only was significant for LMICs 1.31 (95% CI: 1.12, 1.53). Early‐life exposure to animals, that is, dogs and cats, had no association with AR either at the individual or school level.

### 3.4 Multiple logistic regression for school‐level risk factors for adolescents

For the adolescents, use of paracetamol was associated with AR OR: 3.2 (2.32; 4.41) at the school level, with this association also being significant at the individual‐level use OR: 1.91 (95% CI: 1.84, 1.98) (Table 4). Paracetamol use was a consistent risk factor in both HIC and LMIC, OR: 2.06 (95% CI: 1.90, 2.24) and 1.87 (95% CI: 1.79, 1.95), respectively (Table 4; Tables S4 and S7).

**TABLE 4:** Individual‐level data on associations between exposure and allergic rhinitis in adolescents.

Age 13–14, Exposure	GAN fully adjusted^a^		
	All (*n* = 122,170)	LMIC (*n* = 96,397)	HIC (*n* = 25,773)
	OR (95% CI)	OR (95% CI)	OR (95% CI)
More than 1 sibling	1.02 (0.98, 1.06)	1.01 (0.97, 1.06)	1.04 (0.96, 1.13)
More than 2 siblings	NA	NA	NA
Truck traffic	NA	NA	NA
Heavy truck traffic	1.27 (1.23, 1.32)	1.25 (1.20, 1.31)	1.33 (1.23, 1.44)
Fast food	1.05 (1.01, 1.08)	1.05 (1.01, 1.09)	1.03 (0.96, 1.11)
Fast food (excluding burgers)	NA	NA	NA
Meat (frequent)	1.19 (1.15, 1.24)	1.21 (1.16, 1.27)	1.15 (1.07, 1.23)
Television	1.08 (1.03, 1.12)	1.11 (1.05, 1.16)	1.01 (0.93, 1.09)
Computer	1.28 (1.22, 1.34)	1.28 (1.21, 1.35)	1.24 (1.10, 1.39)
Cat	1.15 (1.10, 1.20)	1.17 (1.12, 1.22)	1.08 (0.99, 1.19)
Dog	1.14 (1.10, 1.19)	1.14 (1.09, 1.20)	1.13 (1.04, 1.22)
Paracetamol	1.91 (1.84, 1.98)	1.87 (1.79, 1.95)	2.06 (1.90, 2.24)
Ever smoke	1.37 (1.29, 1.46)	1.37 (1.27, 1.48)	1.37 (1.22, 1.54)

*Note*: NA: collinear with other variables not added to fully adjusted model.

^a^ Adjusted for sex and country income level.

Use of computers for more than an hour daily increased the odds of AR at both the individual and school level, OR: 1.28 (95% CI: 1.22, 1.35) and OR: 2.01 (95% CI: 1.45, 2.79), respectively, and this was consistent for both HIC and LMICs.

Exposure to dogs was an association with at the school‐level OR: 2.30 (1.58, 3.35) and at the individual‐level OR: 1.14 (1.10–1.19). For cats, there was no association at the school‐level OR: 1.03 (0.72, 1.46) but there was an association at the individual‐level OR: 1.15 (1.1, 1.20).

Exposure to heavy truck traffic was associated with increased risk of AR (OR = 1.27; 95% CI: 1.23, 1.32), and on further analysis by country income this association was significant both in HIC OR: 1.33 (95% CI: 1.23, 1.44) and LIMCs OR: 1.25 (95% CI: 1.20, 1.31).

There was no association with ever smoking and AR at school level 1.23 (0.76–1.99), but individual level data found an association with OR: 1.37 (95% CI: 1.29, 1.46) and this was independent of country affluence.

## 4 DISCUSSION

In this GAN Phase I study, we found that the overall global individual prevalence of AR remained relatively stable compared with ISAAC Phase III^14^ in both children and adolescents with 8.5% and 13.3% versus 8.9% and 14.1%, respectively. With regard to the exposures, there was a higher proportion of adolescents with lifestyle‐related risk factors, with almost half consuming fast foods more than once a week and almost three‐quarters (74.7%) using computers more than 1 h per day, which was double that of the children (37.5%). Of concern, almost 10% individual adolescents reported ever smoking, similar to the WHO reported data^17^ We analyzed both individual and school‐level risk factors; as school‐level risk factors are less prone to reverse causation than the individual‐level risk factors, a change in behaviour by a few people with disease will not greatly affect the school‐level prevalence of that risk factor. In contrast, school‐level risk factors on their own are prone to ecological bias. Thus, a similar result at both levels can be interpreted as suggestive evidence against reverse causation.

Paracetamol use in the past 12 months of life was a strong risk factor for AR in both children and adolescents; this was similar to the ISAAC III dataset with OR = 2.02 (95% CI: 1.92, 2.13) for children and OR = 1.76 (95% CI: 1.71, 1.81) for adolescents. Similarly, this finding was consistent for both HIC and LMICs and remained an association at both the individual and school level, therefore providing evidence against reverse causation. This is consistent with animal models and epidemiological data demonstrating a strong association between paracetamol use and AR, with a systematic review and meta‐analysis finding acetaminophen exposure associated with an increased risk of AR (OR = 1.54; 95% CI: 1.41–1.69).^18^, ^19^

Breastfeeding has been found to have a protective effect in other allergic disorders like atopic dermatitis.^20^ A recent systematic review in over 770,000 children found a protective effect of exclusive breastfeeding for more than 6 months for AR reducing the risk by 12%.^21^ Another systematic review found that non‐exclusive breastfeeding for longer than 12 months also added protection against AR.^22^ In the current study, we did not replicate this finding, and breastfeeding ever conferred a risk for AR in HICs at the individual level, but this association was not replicated at the school level, suggesting that there may be reverse causation as mothers who are atopic may breastfeed their children to protect them from other atopic manifestations, such as eczema and asthma. This is a limitation of the cross‐sectional design of the study.

Use of antibiotics in early life has been shown to disrupt the gut microbial community and predispose children to later allergic conditions.^23^ We found an association between AR and early antibiotic use in the first year of life, similar to ISAAC III^14^ (OR = 1.39; 95% CI: 1.03, 1.88). This is consistent with findings by Ni et al in a US‐based study of 2398 children, who found an association between any lifetime use of any antibiotic and AR.^24^

We found a sedentary lifestyle with use of computers more than an hour daily increased the risk of AR in adolescents; this association was significant at both the individual and school level. A previous National Health Survey based study found that children with sleep disturbance due to allergic conditions were more likely to have increased watching of television and video games and consequent lower levels of physical activity^25^ A recent large cluster analysis study comparing two clusters “unhealthy lifestyle and higher moderate‐to‐vigorous activity (MVPA)” compared with “healthier lifestyle with lower MVPA” in 9–11‐year‐old Finnish children found no association between allergies and lifestyle factors^26^

Early‐life exposure to farm animals has been extensively studied in HIC, with strong evidence that exposure to farm animals in the first year of life prevents allergies, including AR.^27^, ^28^, ^29^ The recent data for the Protection against Allergy‐Study in Rural Environments (PASTURE) birth cohort found that exposure to the farming environment in the first 12 months of life confers protection, but this protection is maintained in those who continued to consume unpasteurized milk throughout childhood to maintain the protective effect at age 10 years, suggesting that not only the window of exposure but continued exposure was relevant in conferring protection from allergies^30^ In the current study, we found the opposite, with exposure to farm animals in the first year of life being a risk factor for AR, particularly for children in LMICs. We postulate that potential reasons for this may be, for example, differences in host microbial diversity, dietary practices, animal species, environmental exposures, immunological responses, hygiene practices, and genetic susceptibility. A recent study in young children showed that exposure to traffic‐related air pollution weakened the protective effect of having pets (dogs) against the risk of AR.^31^

The current study has several strengths in that we collected global data in a standardized way from over 200,000 children and adolescents which included both high‐ and low‐ to middle‐income countries. Although there are robust data in HIC settings, the strength in our dataset is from the higher proportion of children and adolescents from LMICs providing much needed data from these settings.

This study's cross‐sectional design restricts the ability to establish temporal relationships between exposures and AR outcomes. Reliance on self‐reported questionnaire data, without confirmation by healthcare professionals, may introduce misclassification bias, which would be applicable at all settings in both HIC and LIMC, except for access to healthcare providers where the diagnosis of AR would be confirmed, but this is mitigated by addition of symptom questions which would classify the individual as having symptoms compatible with AR. Additionally, breastfeeding data lacked granularity regarding timing and duration, limiting interpretation of its potential protective effect. Therefore, these data should be interpreted with caution and is an area that warrants more detailed investigation across diverse resource settings to address this critical issue of diet and its association with AR. Antibiotic exposure was assessed only within the first 12 months of life; later exposures may also influence risk and should be explored in future studies. Our analysis of farming‐related exposures was constrained by the absence of information on residence in farming environments; instead, we captured only contact with farm animals. Additionally, the study design precluded assessment of dose–response relationships and critical exposure windows, which are essential for understanding causality. Finally, due to the multiplicity of exposure variables we analyzed, these were largely exploratory in nature.

In summary, we found the prevalence of AR to be stable in both children and adolescents over a 10‐year period. In the first year of life, exposure to antibiotics and paracetamol increased the risk of AR. Additionally, for adolescents, the use of computers and tobacco smoking conferred increased risk. Exposure to heavy truck traffic was a consistent risk factor of AR in children and adolescents. Finally, exposure to farm animals in early life did not confer protection for AR in children in LMICs, and future longitudinal cohort studies in LMICs are required to further explore this association. These findings highlight the importance of antimicrobial stewardship, clean air policies, and tobacco control as actionable policy implementations for the prevention of AR.

## AUTHOR CONTRIBUTIONS

**Refiloe Masekela:** Acquisition, investigation, data collation, data analysis, writing. **Charlotte E. Rutter:** Formal analysis. **Kevin Mortimer:** Funding acquisition, investigation. **Chen‐Yuan Chiang:** Investigation. **Luis García‐Marcos:** Investigations, funding, writing. **Asma El Sony:** Investigation. **Karen Bissell:** Investigation. **Eamon Ellwood:** Software. **Neil Pearce:** Investigation, funding acquisition. **Innes Asher:** Investigation, writing—original draft. **Phillipa Ellwood:** Investigation. **Nishtha Singh:** Writing. **Anele Khumalo:** Project administration. **Eva Morales:** Analysis, collation, writing.

## FUNDING INFORMATION

This work was supported by the International Union Against Tuberculosis and Lung Disease (The Union); Boehringer Ingelheim New Zealand; AstraZeneca Educational Grant; the National Institute for Health Research; the UK Medical Research Council; the European Research Council; and the Instituto de Salud Carlos III, Spain.

## CONFLICT OF INTEREST STATEMENT

RM reports grant funding and advisory fees from AstraZeneca and Sanofi outside submitted work. KM reports receiving advisory board fees from AstraZeneca outside the submitted work. GBM reports grants and non‐financial support from AstraZeneca and grants from GlaxoSmithKline Australia and Novartis Australia outside the submitted work. All other authors declare no competing interests.

## Supporting information


Tables S1–S7

## Data Availability

The data that support the findings of this study are available from the corresponding author upon reasonable request.
