# Supplementary material for: Environmental risk factors for allergic rhinitis differ by income level: A Global Asthma Network survey
Source: Pediatr Allergy Immunol. 2026 Apr 10;37(4):e70332. doi: 10.1111/pai.70332 (PMC13067801; doi:10.1111/pai.70332)
Supplement: Supplementary file 1 — Tables S1–S7 [file PAI-37-e70332-s001.docx]

**Supplemental Table 1: Variable definition for children 6-7 years**

| Variable | Question | Definition of positive response | Definition of negative response |
| --- | --- | --- | --- |
| AR in past 12 months | Has this child ever had a problem with sneezing or a runny or blocked nose when he / she DID NOT have a cold or the flu? and In the past 12 months, has this child had a problem with sneezing or a runny or blocked nose when he / she DID NOT have a cold or the flu? and In the past 12 months, has this child’s nose problem been accompanied by itchy-watery eyes? | Yes to all | No to any |
| Animals in utero | Did this child’s mother have regular (at least once a week) contact with farm animals (e.g. cattle, pigs, goats, sheep or poultry; use local terminology) while being pregnant with this child? | Yes | No |
| Smoking while pregnant | Did this child’s mother smoke during her pregnancy with this child? | Yes | No |
| Low birthweight | What was the weight of this child when he/she was born? | <2.5kg | >= 2.5kg |
| Paracetamol when young | In the first 12 months of this child’s life, did you usually give paracetamol (use local terminology e.g. Acetaminophen, Panadol, Tylenol) for fever? | Yes | No |
| Antibiotics when young | In the first 12 months of life, did this child have any antibiotics? | Yes | No |
| Breastfed ever | Was this child ever breastfed? | Yes | No |
| Cat when young | Did you have a cat in your home during the first year of this child’s life? | Yes | No |
| Dog when young | Did you have a dog in your home during the first year of this child’s life? | Yes | No |
| Farm animals when young | In this child’s first year of life did this child have regular (at least once a week) contact with farm animals (e.g. cows, cattle, pigs, goats, sheep or poultry; use local terminology)? | Yes | No |
| More than 1 sibling | How many older brothers and/or sisters does this child have? and  How many younger brothers and/or sisters does this child have? | Total number of siblings >=2 | Total number of siblings 0 or 1 |
| More than 2 siblings | How many older brothers and/or sisters does this child have? and  How many younger brothers and/or sisters does this child have? | Total number of siblings >=3 | Total number of siblings <=2 |
| Truck traffic | How often do trucks pass through the street where you live on weekdays? | Seldom (not often) or Frequently through the day or Almost the whole day | Never |
| Heavy truck traffic | How often do trucks pass through the street where you live on weekdays? | Frequently through the day or Almost the whole day | Never or Seldom (not often) |
| Variable | Question | Definition of  Positive response | Definition of negative response |
| Fastfood | In the past 12 months, how often, on average, did this child eat fast food/burgers? | Most or all days or Once or twice per week | Never or only occasionally |
| Fastfood (excl burgers) | In the past 12 months, how often, on average, did this child eat fast food excluding burgers? | Most or all days or Once or twice per week | Never or only occasionally |
| Meat (frequent) | In the past 12 months, how often, on average, did this child eat meat (eg beef, lamb, chicken, pork) | Most or all days | Never or only occasionally or Once or twice per week |
| Television | During a normal week of 7 days, how many hours a day (24 hours) does this child watch television (include DVD’s films, videos)? | 1 hour but less than 3 hours or 3 hours but less than 5 hours or 5 hours or more | Less than 1 hour |
| Computer | During a normal week of 7 days, how many hours a day (24 hours) does this child spend on any of the following: computer (include PlayStation, smartphone, tablet); the internet (include Chat, Facebook, games, Twitter, YouTube) and more? | 1 hour but less than 3 hours or 3 hours but less than 5 hours or 5 hours or more | Less than 1 hour |
| Cat | In the past 12 months, have you had a cat in your home? | Yes | No |
| Dog | In the past 12 months, have you had a dog in your home? | Yes | No |
| Paracetamol | In the past 12 months how often, on average, have you given this child paracetamol (use local terminology e.g. Acetaminophen, Panadol, Pamol, Tylenol) for fever? | At least once a month | Never or At least once a year |

**Supplemental Table 2: Variable definition for adolescents 13-14 years**

| Variable | Question | Definition of positive response | Definition of negative response |
| --- | --- | --- | --- |
| AR in past 12 months | Have you ever had a problem with sneezing or a runny or blocked nose when you DID NOT have a cold or the flu? and In the past 12 months, have you had a problem with sneezing or a runny or blocked nose when you DID NOT have a cold or the flu? and In the past 12 months, has this nose problem been accompanied by itchy-watery eyes? | Yes to all | No to any |
| More than 1 sibling | How many older brothers and/or sisters do you have? and  How many younger brothers and/or sisters do you have? | Total number of siblings >=2 | Total number of siblings 0 or 1 |
| More than 2 siblings | How many older brothers and/or sisters do you have? and  How many younger brothers and/or sisters do you have? | Total number of siblings >=3 | Total number of siblings <=2 |
| Truck traffic | How often do trucks pass through the street where you live on weekdays? | Seldom (not often) or Frequently through the day or Almost the whole day | Never |
| Heavy truck traffic | How often do trucks pass through the street where you live on weekdays? | Frequently through the day or Almost the whole day | Never or Seldom (not often) |
| Fastfood | In the past 12 months, how often, on average, did you eat fast food/burgers? | Most or all days or Once or twice per week | Never or only occasionally |
| Fastfood (excl burgers) | In the past 12 months, how often, on average, did you eat fast food excluding burgers? | Most or all days or Once or twice per week | Never or only occasionally |
| Meat (frequent) | In the past 12 months, how often, on average, did you eat meat (eg beef, lamb, chicken, pork) | Most or all days | Never or only occasionally or Once or twice per week |
| Variable | Question | Definition of positive response | Definition of negative response |
| Television | During a normal week of 7 days, how many hours a day (24 hours) do you watch television (include DVD’s films, videos)? | 1 hour but less than 3 hours or 3 hours but less than 5 hours or 5 hours or more | Less than 1 hour |
| Computer | During a normal week of 7 days, how many hours a day (24 hours) do you spend on any of the following: computer (include PlayStation, smartphone, tablet); the internet (include Chat, Facebook, games, Twitter, YouTube) and more? | 1 hour but less than 3 hours or 3 hours but less than 5 hours or 5 hours or more | Less than 1 hour |
| Cat | In the past 12 months, have you had a cat in your home? | Yes | No |
| Dog | In the past 12 months, have you had a dog in your home? | Yes | No |
| Paracetamol | In the past 12 months how often, on average, have you taken paracetamol (use local terminology e.g. Acetaminophen, Panadol, Pamol, Tylenol) for fever? | At least once a month | Never or At least once a year |
| Ever smoke | In the past, have you smoked tobacco on a daily basis, less than daily, or not at all? | Daily or Less than daily | Not at all |

**Supplemental Table 3: Income levels of data collection countries**

| Country Name | Age 13-14 | Age 6-7 | Income Level |
| --- | --- | --- | --- |
| Argentina | Yes | No | HIC |
| Brazil | Yes | No | LMIC |
| Cameroon | Yes | Yes | LMIC |
| Chile | Yes | No | HIC |
| Costa Rica | Yes | Yes | LMIC |
| Ecaudor | Yes | No | LMIC |
| Greece | Yes | No | HIC |
| Honduras | Yes | Yes | LMIC |
| India | Yes | Yes | LMIC |
| Iran | Yes | Yes | LMIC |
| Kingdom of Saudi Arabia | Yes | Yes | HIC |
| Kosovo | Yes | Yes | LMIC |
| México | Yes | Yes | LMIC |
| New Zealand | Yes | Yes | HIC |
| Nicaragua | Yes | Yes | LMIC |
| Nigeria | Yes | No | LMIC |
| Poland | Yes | No | HIC |
| Russia | Yes | Yes | LMIC |
| South Africa | Yes | No | LMIC |
| Spain | Yes | Yes | HIC |
| Sri Lanka | Yes | Yes | LMIC |
| Sudan | Yes | No | LMIC |
| Syrian Arab Republic | Yes | Yes | LMIC |
| Taiwan | Yes | Yes | HIC |
| Thailand | Yes | Yes | LMIC |

**Supplemental Table 4: School level associations between exposure and allergic rhinoconjuctivitis for children by country income level.**

| **Age 6-7** | Fully adjusted* | | | ISAAC synthesis paper (n=116,863) |
| --- | --- | --- | --- | --- |
|  | All  (n=62,971) | LMIC  (n=46,426) | HIC  (n=16,545) | Fully** |
| Exposure | OR (95% CI) | OR (95% CI) | OR (95% CI) | OR (95% CI) |
| Animals in utero | 1.75 (0.78, 3.94) | 0.97 (0.37, 2.55) | 10.78 (2.28, 51.00) | 1.16 (0.61, 2.20) |
| Smoking while pregnant | 0.87 (0.37, 2.04) | 0.95 (0.18, 4.90) | 0.82 (0.31, 2.17) | NA |
| Low birthweight | 0.70 (0.35, 1.40) | 0.75 (0.34, 1.64) | 0.68 (0.17, 2.82) | 2.59 (1.56, 4.29) |
| Paracetamol when young | 0.92 (0.60, 1.40) | 0.92 (0.54, 1.55) | 0.87 (0.44, 1.71) | 0.99 (0.73, 1.35) |
| Antibiotics when young | 1.84 (1.23, 2.75) | 1.82 (1.09, 3.03) | 1.63 (0.83, 3.18) | 1.39 (1.03, 1.88) |
| Breastfed ever | 0.92 (0.56, 1.53) | 0.73 (0.40, 1.34) | 1.29 (0.54, 3.10) | 0.62 (0.44, 0.88) |
| Cat when young | 1.28 (0.62, 2.63) | 1.46 (0.64, 3.34) | 0.31 (0.06, 1.48) | 1.10 (0.72, 1.68) |
| Dog when young | 1.44 (0.88, 2.34) | 1.73 (0.99, 3.03) | 0.58 (0.20, 1.64) | 0.94 (0.67, 1.31) |
| Animals when young | 0.99 (0.43, 2.26) | 1.56 (0.59, 4.16) | 0.28 (0.05, 1.50) | 1.19 (0.65, 2.18) |
| More than 1 sibling | 1.02 (0.73, 1.43) | 1.16 (0.77, 1.75) | 0.66 (0.35, 1.25) | 0.88 (0.69, 1.12) |
| More than 2 siblings | NA | NA | NA | NA |
| Truck traffic | NA | NA | NA | NA |
| Heavy truck traffic | 1.29 (0.91, 1.82) | 1.21 (0.80, 1.83) | 1.75 (0.93, 3.28) | 0.92 (0.73, 1.16) |
| Fast food | 0.79 (0.54, 1.16) | 0.73 (0.46, 1.16) | 1.15 (0.57, 2.28) | 1.04 (0.82, 1.32) |
| Fast food (excluding burgers) | NA | NA | NA | NA |
| Meat (frequent) | 1.82 (1.28, 2.60) | 2.48 (1.60, 3.83) | 0.81 (0.43, 1.52) | NA |
| Television | 0.91 (0.60, 1.37) | 1.14 (0.67, 1.92) | 0.60 (0.32, 1.14) | 1.45 (1.05, 2.01) |
| Computer | 1.59 (1.11, 2.28) | 1.36 (0.87, 2.11) | 2.05 (1.08, 3.88) | NA |
| Cat | 0.45 (0.25, 0.82) | 0.31 (0.16, 0.61) | 3.79 (0.98, 14.68) | NA |
| Dog | 1.52 (0.97, 2.38) | 1.57 (0.93, 2.64) | 1.55 (0.59, 4.09) | NA |
| Paracetamol | 2.11 (1.36, 3.26) | 2.41 (1.44, 4.02) | 1.72 (0.72, 4.12) | 2.04 (1.43, 2.89) |

*Adjusted for sex and country income level, ** adjusted for sex, country income level, maternal education, paternal and maternal tobacco use and open fire cooking, NA: collinear with other variable not added to fully adjusted model.

**Supplemental Table 5: School level associations between exposures and risk factors for allergic rhinoconjuctivitis for adolescents by country income level**

| **Age 13-14** | Fully adjusted* | | | ISAAC synthesis paper |
| --- | --- | --- | --- | --- |
|  | All  (n=122,170) | LMIC  (n=96,397) | HIC  (n=25,773) | Fully**  (n=224,436) |
| Exposure | OR (95% CI) | OR (95% CI) | OR (95% CI) | OR (95% CI) |
| More than 1 sibling | 1.02 (0.79, 1.34) | 1.12 (0.82, 1.54) | 0.68 (0.43, 1.08) | 0.93 (0.75, 1.15) |
| More than 2 siblings | NA | NA | NA | NA |
| Truck traffic | NA | NA | NA | NA |
| Heavy truck traffic | 1.43 (1.06, 1.92) | 1.37 (0.97, 1.94) | 1.61 (0.90, 2.90) | 1.16 (0.94, 1.44) |
| Fast food | 0.88 (0.67, 1.16) | 0.83 (0.59, 1.16) | 1.27 (0.75, 2.14) | 1.24 (1.02, 1.51) |
| Fast food (excluding burgers) | NA | NA | NA | NA |
| Meat (frequent) | 1.88 (1.41, 2.51) | 1.44 (1.01, 2.08) | 3.81 (2.37, 6.12) | NA |
| Television | 0.75 (0.53, 1.06) | 0.78 (0.51, 1.19) | 0.67 (0.37, 1.22) | 1.20 (0.86, 1.68) |
| Computer | 2.01 (1.45, 2.79) | 2.06 (1.40, 3.03) | 2.19 (1.03, 4.62) | NA |
| Cat | 1.03 (0.72, 1.46) | 0.87 (0.57, 1.32) | 1.68 (0.87, 3.25) | NA |
| Dog | 2.30 (1.58, 3.35) | 2.95 (1.87, 4.65) | 1.03 (0.54, 1.97) | NA |
| Paracetamol | 3.20 (2.32, 4.41) | 3.52 (2.43, 5.11) | 2.10 (1.11, 3.97) | 3.48 (2.66, 4.56) |
| Ever smoke | 1.23 (0.76, 1.99) | 1.01 (0.56, 1.81) | 2.47 (1.06, 5.79) | NA |

*Adjusted for sex and country income level; ** adjusted for sex, country income level, maternal education, paternal and maternal tobacco use and open fire cooking, NA: collinear with other variable not added to fully adjusted model.

**Supplemental Table 6: individual level associations between exposure and allergic rhinoconjuctivitis for children by country income level comparing ISAACIII and GAN I.**

| Age 6-7 | GAN Fully adjusted* | | | ISAAC synthesis paper |
| --- | --- | --- | --- | --- |
|  | All  (n=62,971) | LMIC  (n=46,426) | HIC  (n=16,545) | Fully adjusted**  (n=116,863) |
| Exposure | OR (95% CI) | OR (95% CI) | OR (95% CI) | OR (95% CI) |
| Animals in utero | 1.18 (1.04, 1.34) | 1.22 (1.05, 1.42) | 1.12 (0.88, 1.41) | 1.18 (1.07, 1.30) |
| Smoking while pregnant | 1.16 (1.01, 1.33) | 1.63 (1.31, 2.04) | 0.99 (0.82, 1.18) | NA |
| Low birthweight | 1.08 (0.97, 1.21) | 1.08 (0.93, 1.24) | 1.09 (0.90, 1.32) | 1.04 (0.96, 1.13) |
| Paracetamol when young | 1.13 (1.05, 1.22) | 1.06 (0.95, 1.18) | 1.22 (1.09, 1.36) | 1.39 (1.32, 1.47) |
| Antibiotics when young | 1.67 (1.56, 1.78) | 1.86 (1.71, 2.02) | 1.38 (1.24, 1.54) | 1.58 (1.51, 1.66) |
| Breastfed ever | 1.11 (1.01, 1.22) | 1.05 (0.94, 1.18) | 1.19 (1.02, 1.38) | 1.00 (0.95, 1.06) |
| Cat when young | 1.06 (0.95, 1.19) | 1.14 (1.00, 1.29) | 0.84 (0.67, 1.07) | 1.09 (1.01, 1.16) |
| Dog when young | 1.14 (1.06, 1.23) | 1.19 (1.10, 1.30) | 0.95 (0.80, 1.12) | 1.07 (1.01, 1.12) |
| Animals when young | 1.24 (1.09, 1.42) | 1.31 (1.12, 1.53) | 1.11 (0.86, 1.44) | 1.07 (0.98, 1.17) |
| More than 1 sibling | 0.91 (0.85, 0.98) | 0.92 (0.85, 0.99) | 0.88 (0.76, 1.01) | 0.98 (0.94, 1.03) |
| More than 2 siblings | NA | NA | NA | NA |
| Truck traffic | NA | NA | NA | NA |
| Heavy truck traffic | 1.31 (1.23, 1.40) | 1.35 (1.25, 1.46) | 1.23 (1.09, 1.40) | 1.17 (1.12, 1.22) |
| Fast food | 0.96 (0.89, 1.02) | 0.93 (0.86, 1.01) | 1.00 (0.89, 1.12) | 0.99 (0.94, 1.03) |
| Fast food (excluding burgers) | NA | NA | NA | NA |
| Meat (frequent) | 1.16 (1.09, 1.24) | 1.28 (1.18, 1.39) | 0.97 (0.87, 1.08) | NA |
| Television | 0.97 (0.90, 1.04) | 0.97 (0.88, 1.06) | 0.97 (0.86, 1.09) | 0.93 (0.88, 0.98) |
| Computer | 1.09 (1.02, 1.16) | 1.12 (1.03, 1.21) | 1.03 (0.92, 1.15) | NA |
| Cat | 0.93 (0.84, 1.02) | 0.92 (0.83, 1.03) | 1.01 (0.81, 1.25) | NA |
| Dog | 0.98 (0.91, 1.06) | 0.99 (0.91, 1.08) | 1.01 (0.86, 1.19) | NA |
| Paracetamol | 2.03 (1.89, 2.18) | 1.98 (1.83, 2.15) | 2.17 (1.89, 2.51) | 2.02 (1.92, 2.13) |

*Adjusted for sex and country income level, ** adjusted for sex, country income level, maternal education, paternal and maternal tobacco use and open fire cooking, NA: collinear with other variable not added to fully adjusted model.

**Supplemental Table 7: Individual level associations between exposures and risk factors for**

**allergic rhinoconjuctivitis for adolescents by country income level comparing ISAAC III and GAN I**

*Adjusted for sex and country income level, ** adjusted for sex, country income level, maternal education, paternal and maternal tobacco use and open fire cooking, NA: collinear with other variable not added to fully adjusted model.

| Age 13-14 | GAN Fully adjusted | | | ISAAC synthesis paper |
| --- | --- | --- | --- | --- |
|  | All  (n=122,170) | LMIC  (n=96,397) | HIC  (n=25,773) | Fully*  (n=224,436) |
| Exposure | OR (95% CI) | OR (95% CI) | OR (95% CI) | OR (95% CI) |
| More than 1 sibling | 1.02 (0.98, 1.06) | 1.01 (0.97, 1.06) | 1.04 (0.96, 1.13) | 1.04 (1.01, 1.07) |
| More than 2 siblings | NA | NA | NA | NA |
| Truck traffic | NA | NA | NA | NA |
| Heavy truck traffic | 1.27 (1.23, 1.32) | 1.25 (1.20, 1.31) | 1.33 (1.23, 1.44) | 1.23 (1.20, 1.26) |
| Fast food | 1.05 (1.01, 1.08) | 1.05 (1.01, 1.09) | 1.03 (0.96, 1.11) | 1.06 (1.03, 1.08) |
| Fast food (excluding burgers) | NA | NA | NA | NA |
| Meat (frequent) | 1.19 (1.15, 1.24) | 1.21 (1.16, 1.27) | 1.15 (1.07, 1.23) | NA |
| Television | 1.08 (1.03, 1.12) | 1.11 (1.05, 1.16) | 1.01 (0.93, 1.09) | 1.01 (0.97, 1.05) |
| Computer | 1.28 (1.22, 1.34) | 1.28 (1.21, 1.35) | 1.24 (1.10, 1.39) | NA |
| Cat | 1.15 (1.10, 1.20) | 1.17 (1.12, 1.22) | 1.08 (0.99, 1.19) | NA |
| Dog | 1.14 (1.10, 1.19) | 1.14 (1.09, 1.20) | 1.13 (1.04, 1.22) | NA |
| Paracetamol | 1.91 (1.84, 1.98) | 1.87 (1.79, 1.95) | 2.06 (1.90, 2.24) | 1.76 (1.71, 1.81) |
| Ever smoke | 1.37 (1.29, 1.46) | 1.37 (1.27, 1.48) | 1.37 (1.22, 1.54) | NA |
